# Interplay of IL‐6, GDF‐15 and Sarcopenia in Patients With Bladder Cancer Undergoing Radical Cystectomy and Its Implications on Survival

**DOI:** 10.1002/jcsm.70146

**Published:** 2025-12-11

**Authors:** Simon U. Engelmann, Felix Kasparbauer, Christoph Pickl, Francesco Del Giudice, Maximilian Haas, Emily Rinderknecht, Peter J. Siska, Renate Pichler, Christoph Niessen, Maximilian Burger, Miodrag Gužvić, Roman Mayr

**Affiliations:** ^1^ Department of Urology, St. Josef Medical Center University of Regensburg Regensburg Germany; ^2^ Department of Maternal‐Infant and Urological Sciences Sapienza Rome University Rome Italy; ^3^ Department of Internal Medicine III University Hospital Regensburg Regensburg Germany; ^4^ Department of Urology, Comprehensive Cancer Center Innsbruck Medical University of Innsbruck Innsbruck Austria; ^5^ Department of Radiology St. Josef Medical Center Regensburg Germany

**Keywords:** biomarkers, bladder cancer, GDF‐15, interleukin 6, sarcopenia, urothelial cancer

## Abstract

**Background:**

Sarcopenia has emerged as a significant predictor of adverse outcomes in cancer. Specifically, this is also true for patients with bladder cancer undergoing radical cystectomy (RC). This retrospective study investigates the roles of the biomarkers interleukin‐6 (IL‐6) and growth differentiation factor‐15 (GDF‐15), in the context of sarcopenia, assessing their impact on oncological and survival outcomes.

**Methods:**

Preoperative serum IL‐6 and GDF‐15 levels were analysed in 179 patients undergoing RC. Their association with sarcopenia, adverse pathological features and survival outcomes was investigated.

**Results:**

Elevated IL‐6 and GDF‐15 levels were significantly correlated with the presence of sarcopenia (*p* = 0.04 and *p* = 0.03, respectively). IL‐6 and GDF‐15 levels in serum showed a positive correlation (Spearman *r* = 0.45, 95%CI 0.32–0.56, *p* < 0.01). Higher IL‐6 and GDF‐15 levels were also associated with higher tumour stages (both *p* < 0.01), positive lymph nodes (*p* = 0.02 and *p* < 0.01) and unfavourable surgical margins (both *p* < 0.01). Patients with both sarcopenia and high IL‐6 or GDF‐15 levels exhibited significantly worse overall survival and cancer‐specific survival in multivariate Cox regression analysis.

**Conclusions:**

These findings highlight the interplay between IL‐6, GDF‐15, sarcopenia and tumour progression, suggesting that IL‐6 and GDF‐15 may serve as valuable prognostic biomarkers and potential therapeutic targets. Further research is warranted to explore targeted therapeutic strategies aimed at mitigating sarcopenia and systemic inflammation in this patient population.

## 1 Introduction

Patients with bladder cancer (BC) undergoing radical cystectomy (RC) face numerous factors that significantly influence their surgical outcomes and survival rates. Among these, sarcopenia—a condition marked by the progressive depletion of skeletal muscle mass and function—has emerged as a critical predictor of negative outcomes. Research indicates that sarcopenia correlates with elevated rates of postoperative complications, extended hospitalisations and diminished overall survival (OS) and cancer‐specific survival (CSS) [1, 2, 3, 4]. Furthermore, sarcopenia is associated with reduced chemotherapy tolerance and an increased susceptibility to perioperative complications, such as infections and delayed wound healing, thereby further impacting survival rates [5].

Inflammatory biomarkers play a pivotal role in the pathophysiology of sarcopenia and BC progression, with interleukin‐6 (IL‐6) being a key contributor. IL‐6 promotes tumour progression by fostering a pro‐inflammatory microenvironment that supports angiogenesis, tumour cell survival and metastasis [6]. Elevated IL‐6 levels have been shown to correlate with adverse pathological features such as muscle invasion, lymph node metastasis and increased recurrence rates in patients with BC undergoing cystectomy [7, 8]. In a preliminary study, we were able to identify serum IL‐6 as a prognostic marker for overall and CSS in this cohort [8]. However, further investigations are necessary to investigate associations with sarcopenia. It is known that IL‐6 accelerates muscle wasting, exacerbating sarcopenia and creating a vicious cycle that further compromises patient recovery and survival [9].

Another critical biomarker implicated in cancer‐related sarcopenia is growth differentiation factor‐15 (GDF‐15). GDF‐15, a member of the transforming growth factor‐beta (TGF‐β) superfamily, is a stress‐responsive cytokine frequently elevated in various cancers, including BC [10]. GDF‐15 has been linked to tumour progression, immune modulation and systemic metabolic alterations that contribute to muscle wasting [11]. High serum GDF‐15 levels are associated with poor prognosis in patients with cancer due to their role in inducing anorexia, weight loss and muscle degradation, further worsening sarcopenia and diminishing physical resilience [12]. GDF‐15‐mediated catabolic signalling promotes skeletal muscle atrophy through pathways that enhance proteolysis and suppress protein synthesis, exacerbating muscle loss in patients undergoing RC [13].

The interplay between IL‐6, GDF‐15 and sarcopenia suggests a complex network of inflammatory and metabolic processes that may act synergistically to worsen outcomes in patients with BC. Both IL‐6 and GDF‐15 are involved in cancer‐related cachexia, with their elevated levels predicting more severe muscle wasting, reduced physical function and diminished ability to withstand the stresses of surgery and cancer treatment. Given their combined impact, these biomarkers may serve as valuable prognostic indicators and potential therapeutic targets to mitigate the detrimental effects of sarcopenia and systemic inflammation.

Understanding the roles of IL‐6 and GDF‐15 in patients with BC undergoing RC is crucial for developing personalised treatment plans aimed at improving survival and quality of life. Targeted interventions, including prehabilitation strategies such as structured exercise programs, nutritional optimisation and anti‐inflammatory therapies, may help counteract muscle wasting and systemic inflammation. By elucidating the combined influence of IL‐6, GDF‐15 and sarcopenia on perioperative outcomes and long‐term survival, this study aims to enhance prognostication, optimise preoperative preparation and refine postoperative care strategies for patients with BC.

## 2 Patients and Methods

### 2.1 Study Design and Patient Selection

After obtaining informed consent, preoperative serum samples were prospectively collected from patients undergoing RC for BC between September 2019 and September 2022 in this single‐centre study. Serum samples were drawn preoperatively on the day of surgery, prior to the induction of anaesthesia, as previously described [8]. Out of 213 patients who underwent RC for BC during this period, 34 were excluded from the study cohort due to missing preoperative serum samples, missing preoperative computed tomography (CT) or incomplete follow‐up information, resulting in a final cohort of 179 patients (84%).

### 2.2 Clinical Data Acquisition

Clinicopathologic characteristics were gathered retrospectively. Follow‐up data were obtained through hospital records and consultations with local urologists and general practitioners. OS was defined as the duration from the date of RC to death from any cause, while CSS was defined as the interval from RC to death specifically attributable to BC progression or metastasis.

### 2.3 Measurement of IL‐6 and GDF‐15 in Serum

IL‐6 was measured using an IL‐6 ELISA assay (Human IL‐6 Quantikine HS ELISA, R&D systems), as previously described [8]. As commonly used in clinical laboratories and research studies, a cutoff of 7 pg/mL was used to distinguish between low and high IL‐6 concentrations in serum. Depending on the disease investigated, cutoff values of serum IL‐6 used in the literature vary. Mostly, median IL‐6 levels of individual study cohorts are used [6, 14, 15, 16]. To bring unity and comparability into this field of research, we chose the standard reference range of 7 pg/mL as a cutoff for our study [17, 18, 19]. GDF‐15 was measured using a GDF‐15 ELISA assay (Human GDF‐15 Immunoassay Quantikine EILSA, R&D systems). As determined by Kamper et al. a cutoff of 1541 pg/mL was used to distinguish between low and high GDF‐15 concentrations in serum [11].

### 2.4 Measurement of Sarcopenia

Sarcopenia was determined by measuring skeletal muscle index (SMI) in preoperative CT scans of the abdomen, at the height of the third lumbar vertebra (L3) using Osirix DICOM viewer software (OsiriX MD version 13.0.0, Pixmeo, Geneva, Switzerland) as previously described [2, 20]. Measurements were performed on two consecutive transversal CT images at the height of L3 with both transverse processes visible. The mean measurements were used for further calculations and analyses. Skeletal muscle was identified as Hounsfield units (HUs) of −29 to +150 [21]. The presence of sarcopenia was defined after Martin et al. [20]. Martin et al. define the presence of sarcopenia as SMI < 43 cm^2^/m^2^ if BMI < 25 kg/m^2^ or SMI < 53 cm^2^/m^2^ if BMI ≥ 25 kg/m^2^ for males and SMI < 41 cm^2^/m^2^ for females.

### 2.5 Statistical Analysis

Frequencies are presented as absolute numbers and percentages. Continuous data are presented as median with interquartile range (IQR). After testing for normal distribution, differences between groups were analysed using the Mann–Whitney *U* test for dichotomous parameters and the Kruskal–Wallis test for categorical data. Chi‐squared statistic and Fisher's exact test were used to determine differences between risk groups with respect to categorical variables. Kaplan–Meier curves were used to illustrate OS and CSS. Statistical analysis was performed using the SPSS software (version 29.0; SPSS Inc., Chicago, Illinois, United States). Graphs were created using GraphPad Prism, version 10.2.1 (GraphPad Software; San Diego, California, United States).

## 3 Results

### 3.1 Serum IL‐6 and Sarcopenia

**FIGURE 1:**
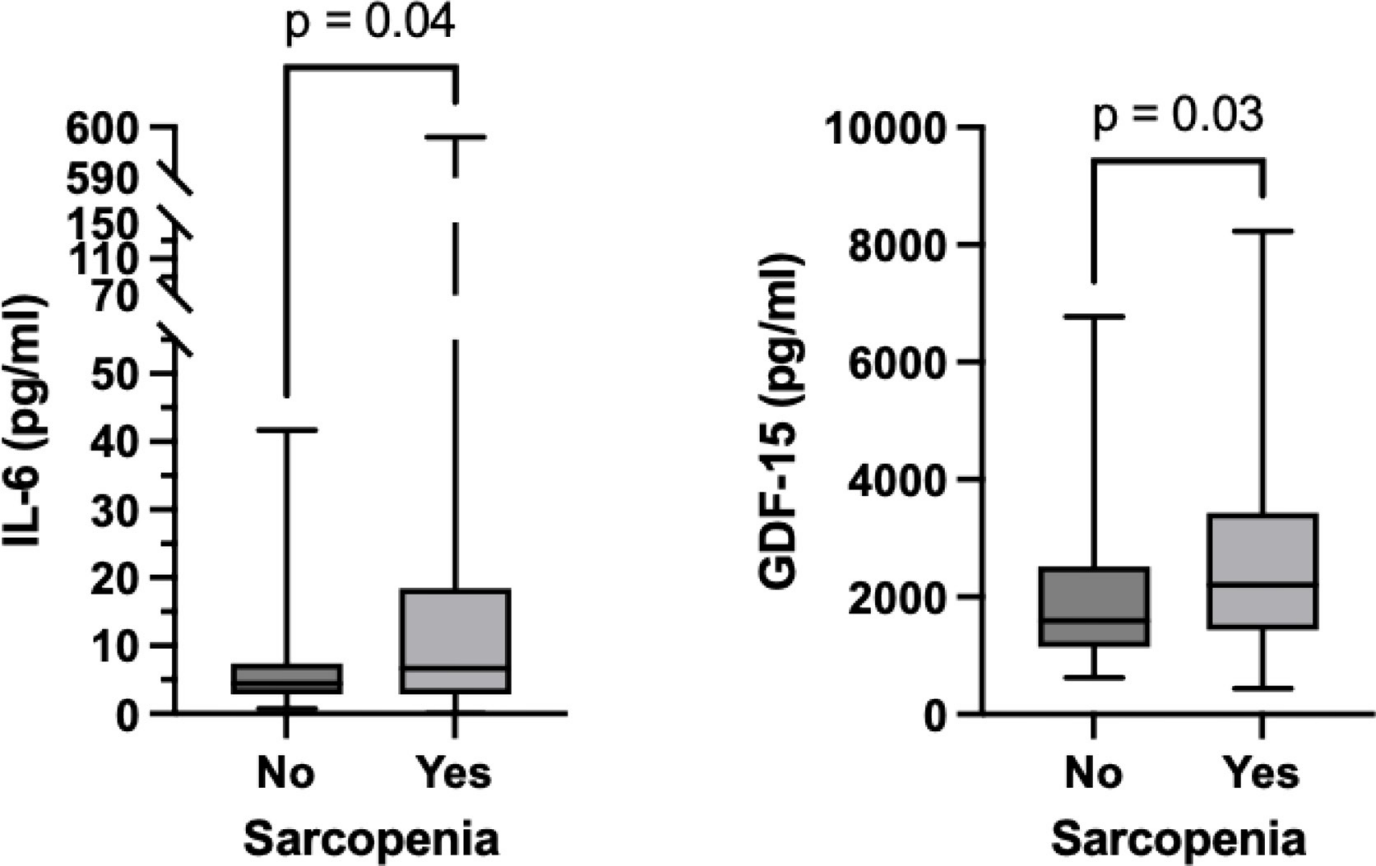
Box plots showing IL‐6 and GDF15 serum values in patients with or without sarcopenia.

Median IL‐6 serum level for the entire study cohort was 5.4 pg/mL (IQR 2.9–13.4). Age (> 70 years), higher ASA score and sarcopenia are preoperative characteristics that were associated with higher IL‐6 serum levels (all *p* < 0.05; Table 1). Unfavourable pathologic characteristics such as higher tumour stage, positive lymph nodes and positive surgical margins were also associated with higher IL‐6 serum levels (all *p* < 0.05; Table 1). Higher IL‐6 serum levels were found in patients with sarcopenia, opposed to patients without sarcopenia (*p* = 0.04; Figure 1). IL‐6, being an inflammatory marker, was positively correlated with CRP (Spearman's *r* = 0.65, 95%CI 0.55–0.73, *p* < 0.01). In univariate logistic regression, high IL‐6 was found to be a predictor of sarcopenia (OR 2.32, 95%CI 1.18–4.54, *p* = 0.02; Table S4). In multivariate analysis, high IL‐6 as an independent predictor for sarcopenia was not quite statistically significant (OR 1.84, 95%CI 0.93–3.65, *p* = 0.08; Table 6). Elevated CRP (> 0.5 mg/dL) was not found to be a predictor of sarcopenia (OR 1.09, 95%CI 0.56–2.14, *p* = 0.79). Detailed characteristics found in patients with and without sarcopenia are shown in Table S3. Patients with sarcopenia more commonly had higher tumour stages at cystectomy (Figure S2).

**TABLE 1:** Patient characteristics and median serum IL‐6 and GDF15 levels associated with different variables.

Characteristic	*n* (%)	Median IL‐6 pg/mL (IQR)	*p* ^a^	Median GDF15 pg/mL (IQR)	*p* ^a^
Age					
≤ 70 years	96 (53.6)	4.44 (2.39–11.19)	< 0.01	1907 (1115–2734)	0.02
> 70 years	83 (46.4)	6.65 (3.81–18.57)		2083 (1489–3500)	
Gender			0.54		0.74
Male	131 (73.2)	5.38 (2.92–11.22)		1996 (1289–2920)	
Female	48 (26.8)	5.89 (2.66–22.96)		2084 (1199–3431)	
ASA score			0.04		< 0.01
1	3 (1.7)	1.59 (1.45–n/a)		1044 (n/a)	
2	70 (39.1)	3.87 (2.15–8.24)		1543 (1031–2599)	
3	97 (54.2)	5.52 (3.33–17.59)		2107 (1460–3220)	
4	9 (5.0)	23.48 (10.02–72.4)		5038 (3212–6455)	
Smoking			0.33		0.04
Yes	97 (54.2)	4.45 (2.72–13.27)		1844 (1181–2888)	
No	82 (45.8)	6.04 (3.01–14.44)		2105 (1486–3626)	
Neoadjuvant chemotherapy			0.59		0.03
Yes	51 (28.7)	6.23 (2.77–9.85)		2391 (1607–3412)	
No	127 (71.3)	4.74 (2.89–15.14)		1844 (1152–3107)	
Tumour stage at cystectomy			< 0.01		< 0.01
pT0	27 (15.1)	2.71 (1.95–6.53)		1489 (988–2481)	
pTa, pT1, pTis	20 (11.2)	4.06 (2.15–7.14)		1590 (1201–2304)	
pT2	33 (18.4)	3.40 (2.11–5.60)		1527 (1208–2430)	
pT3	67 (37.4)	7.93 (3.89–18.46)		2406 (1454–3253)	
pT4	32 (17.9)	13.94 (5.06–38.29)		2574 (1891–4508)	
Nodal stage			0.02		< 0.01
N0	134 (74.9)	4.50 (2.64–10.90)		1860 (1182–2690)	
N+	45 (25.1)	7.93 (3.94–18.51)		2827 (1590–4634)	
Surgical margins			< 0.01		< 0.01
R0	133 (74.3)	4.42 (2.67–9.79)		1857 (1231–2855)	
R+	43 (24.0)	13.14 (4.69–26.94)		2735 (1678–4712)	
Rx	3 (1.7)	1.35 (n/a)		1183 (n/a)	
Sarcopenia (Martin et al.)			0.04		0.03
No	57 (31.8)	4.45 (2.83–7.40)		1590 (1146–2523)	
Yes	122 (68.2)	6.67 (2.83–18.45)		2208 (1435–3437)	
High GDF‐15 (cutoff: 1541 pg/mL)			< 0.01		—
Yes	115 (64.2)	7.01 (3.83–18.46)		—	
No	64 (35.8)	3.23 (1.95–7.03)		—	
High IL‐6 (cutoff: 7 pg/mL)			—		< 0.01
Yes	74 (41.3)	—		2577 (1631–4101)	
No	105 (58.7)	—		1678 (1179–2519)	

^a^ Differences between groups were analysed using the Mann–Whitney *U* test for dichotomous parameters and the Kruskal–Wallis test for categorical data.

### 3.2 Serum GDF‐15 and Sarcopenia

Median GDF‐15 serum level for the entire study cohort was 1996 pg/mL (IQR 1247–3198). Age (< 70 years, *p* = 0.02), higher ASA score (*p* < 0.01), neoadjuvant chemotherapy (*p* = 0.03), and sarcopenia (*p* = 0.03) were associated with higher levels of GDF‐15 (Table 1). Former or current smokers had lower GDF‐15 serum levels (*p* = 0.04; Table 1). Unfavourable pathologic characteristics including higher tumour stage, positive lymph nodes and positive surgical margins were all associated with higher levels of GDF‐15 (all *p* < 0.01). Patients with high IL‐6 levels in serum also had higher GDF‐15 levels in serum (*p* < 0.01; Table 1). In univariate logistic regression, high GDF‐15 was found to be a predictor of sarcopenia (OR 2.07, 95%CI 1.08–3.95, *p* = 0.03; Table S4). In multivariate analysis, high GDF‐15 was found to be an independent predictor for sarcopenia, although not quite statistically significant (OR 1.99, 95%CI 1.00–3.96, *p* = 0.05; Table 6). Patients with sarcopenia had higher levels of GDF‐15 in serum (*p* = 0.03; Figure 1). GDF‐15 and IL‐6 showed a moderate significant positive correlation (Spearman *r* = 0.45, 95%CI 0.32–0.56, *p* < 0.01; Figure 1A).

### 3.3 Survival

**FIGURE 2:**
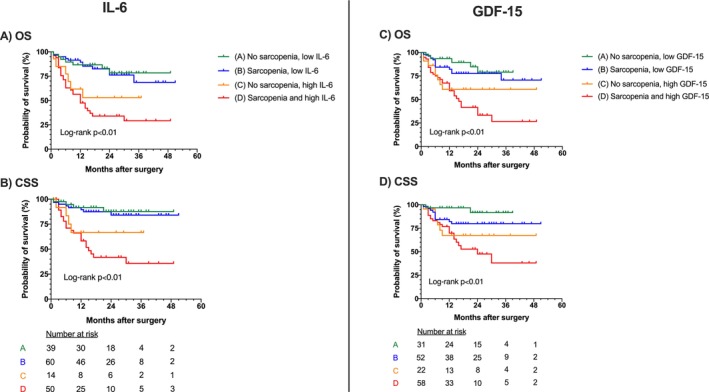
Kaplan–Meier curves showing overall and cancer‐specific survival in different risk groups combining sarcopenia and serum biomarkers.

In total, 65 (36.3%) patients died due to any cause during follow‐up. Forty‐six (25.7%) patients died because of BC. Both high IL‐6 and GDF‐15 in serum, as well as sarcopenia, are independent and combined risk factors in patients undergoing RC for BC. Table 2 shows different risk groups related to the presence of high serum IL‐6 and/or sarcopenia, and their association with histopathological characteristics and survival. The patient group with both high serum IL‐6 and sarcopenia had unfavourable tumour stages and surgical margins, as well as shorter OS and CSS, as compared with other groups (all *p* < 0.01; Table 2; Table S1). Similarly, Table 3 shows risk groups constituted of the presence of high GDF‐15 in serum and/or sarcopenia. Patients with both sarcopenia and high GDF‐15 had higher tumour stages (*p* < 0.01), more often positive surgical margins (*p* < 0.01) and inferior OS (*p* < 0.01) and CSS (*p* = 0.02). After categorising patients into groups by the presence of IL‐6 or GDF‐15 and sarcopenia, significant differences in OS and CSS could be shown between these groups (Figure 2A–D; log‐rank in all cases *p* < 0.01). These findings were confirmed and specified in multivariate Cox regression analysis, where the groups containing patients with high IL‐6 and sarcopenia or patients with high GDF‐15 and sarcopenia both were independently at risk for shorter OS and CSS (OS sarcopenia and high IL‐6: HR 3.99, 95%CI 1.81–8.81, *p* < 0.01; CSS sarcopenia and high IL‐6: HR 4.78, 95%CI 1.79–12.77, *p* < 0.01; OS sarcopenia and high GDF‐15: HR 2.97, 95%CI 1.15–7.70, *p* = 0.03; CSS sarcopenia and high GDF‐15: HR 3.43, 95%CI 1.02–11.56, *p* = 0.047; Tables S5 and S6). Multivariate Cox regression analysis showed that high IL‐6 and high GDF‐15 are both independent predictors of OS and CSS (IL‐6 OS: HR 3.49, 95%CI 2.00–6.08, *p* < 0.001; IL‐6 CSS: HR 4.14, 95%CI 2.10–8.14, *p* < 0.001; GDF‐15 OS: HR 2.30, 95%CI 1.20–4.40, *p* = 0.01; GDF‐15 CSS: HR 2.23, 95%CI 1.04–4.80, *p* = 0.04; Tables 4 and 5). High CRP was also found to be a predictor of OS and CSS (OS: HR 3.27, 95%CI 1.85–5.79, *p* < 0.001; CSS: HR 5.29, 95%CI 2.51–11.15, *p* < 0.001). Sarcopenia was not significantly verified as an independent predictor for OS and CSS in our preoperative multivariate Cox regression model (OS: HR 1.74, 95%CI 0.95–3.18, *p* = 0.07; CSS: HR 1.90, 95%CI 0.91–3.98, *p* = 0.09; Tables 4 and 5).

**TABLE 2:** Different risk groups separated by sarcopenia and high IL‐6 in serum and the distribution of tumour characteristics and survival.

Characteristic	No sarcopenia, low IL‐6	Sarcopenia, low IL‐6	No sarcopenia, high IL‐6	Sarcopenia, high IL‐6	*p*
Tumour stage at cystectomy					< 0.01*
pT0	11 (26.8)	10 (15.6)	2 (12.5)	3 (5.2)	
pTa, pT1, pTis	8 (19.5)	7 (10.9)	3 (18.8)	3 (5.2)	
pT2	10 (24.4)	19 (29.7)	1 (6.3)	3 (5.2)	
pT3	10 (24.4)	21 (32.8)	8 (50)	28 (48.3)	
pT4	2 (4.9)	7 (10.9)	2 (12.5)	21 (36.2)	
Surgical margins					< 0.01**
R0	37 (90.2)	51 (79.7)	11 (68.7)	34 (58.6)	
R+	4 (9.8)	10 (15.6)	5 (31.3)	24 (41.4)	
Rx	—	3 (4.7)	—	—	
Overall death					< 0.01**
No	33 (80.5)	50 (78.1)	10 (62.5)	21 (36.2)	
Yes	8 (19.5)	14 (21.9)	6 (37.5)	37 (63.8)	
Cancer‐specific death					< 0.01**
No	36 (87.8)	55 (85.9)	12 (75)	30 (51.7)	
Yes	5 (12.2)	9 (14.1)	4 (25)	28 (48.3)	

*Chi‐squared statistic was used to determine differences between risk groups with respect to categorical variables.

**Fisher's exact was used to determine differences between risk groups with respect to categorical variables.

**TABLE 3:** Different risk groups separated by sarcopenia and high GDF‐15 in serum and the distribution of tumour characteristics and survival.

Characteristic	No sarcopenia, low GDF‐15	Sarcopenia, low GDF‐15	No sarcopenia, high GDF‐15	Sarcopenia, high GDF‐15	*p*
Tumour stage at cystectomy					< 0.01*
pT0	10 (37.0)	4 (10.8)	3 (10.0)	9 (10.6)	
pTa, pT1, pTis	3 (11.1)	7 (18.9)	8 (26.7)	3 (3.5)	
pT2	5 (18.5)	12 (32.4)	6 (20.0)	10 (11.8)	
pT3	7 (25.9)	10 (27.0)	11 (36.7)	39 (45.9)	
pT4	2 (7.4)	4 (10.8)	2 (6.7)	24 (28.2)	
Surgical margins					< 0.01**
R0	23 (85.2)	29 (78.4)	25 (83.3)	56 (65.9)	
R+	4 (14.8)	5 (13.5)	5 (16.7)	29 (34.1)	
Rx	—	3 (8.1)	—	—	
Overall death					< 0.01**
No	22 (81.5)	29 (78.4)	21 (70.0)	42 (49.4)	
Yes	5 (18.5)	8 (21.6)	9 (30.0)	43 (50.6)	
Cancer‐specific death					0.02**
No	24 (88.9)	31 (83.8)	24 (80.0)	54 (63.5)	
Yes	3 (11.1)	6 (16.2)	6 (20.0)	31 (36.5)	

*Chi‐squared statistic was used to determine differences between risk groups with respect to categorical variables.

**Fisher's exact was used to determine differences between risk groups with respect to categorical variables.

**TABLE 4:** Assessing biomarkers and sarcopenia in preoperative multivariate cox regression model for overall survival including age, clinical tumour stage and radiologically suspected nodal stage prior to cystectomy.

Variable	Model 1 including IL‐6			Model 2 including GDF‐15			Model 3 including sarcopenia		
	HR	(95%CI)	*p*	HR	(95%CI)	*p*	HR	(95%CI)	*p*
Age (years, continuous)	1.03	1.00–1.05	0.05	1.02	1.00–1.05	0.08	1.03	1.0–1.06	0.06
Clinical tumour stage prior to Cx (Ref. < cT2)									
cT2	1.03	0.41–2.63	0.94	1.26	0.50–3.19	0.63	1.50	0.60–3.74	0.38
cT3	1.54	0.52–4.61	0.44	1.62	0.54–4.87	0.39	2.30	0.79–6.75	0.13
cT4	1.59	0.50–5.01	0.43	2.90	0.97–8.70	0.06	3.93	1.34–11.52	0.01
Radiologic suspected N+ prior Cx (Ref. N0)	1.31	0.92–1.86	0.13	1.23	0.86–1.76	0.26	1.11	0.79–1.56	0.56
High IL‐6 (Ref. low IL‐6 < 7 pg/mL)	3.49	2.00–6.08	< 0.001	—	—	—	—	—	—
High GDF15 (Ref. low GDF15 < 1542 pg/mL)	—	—	—	2.30	1.20–4.40	0.01	—	—	—
Sarcopenia (Martin et al. Ref. no)	—	—	—	—	—	—	1.74	0.95–3.18	0.07

**TABLE 5:** Assessing biomarkers and sarcopenia in preoperative multivariate Cox regression model for cancer‐specific survival including age, clinical tumour stage and radiologically suspected nodal stage prior to cystectomy.

Variable	Model 1 including IL‐6			Model 2 including GDF‐15			Model 3 including sarcopenia		
	HR	(95%CI)	*p*	HR	(95%CI)	*p*	HR	(95%CI)	*p*
Age (years, continuous)	1.03	1.00–1.06	0.10	1.03	0.99–1.06	0.13	1.03	0.99–1.06	0.12
Clinical tumour stage prior to Cx (Ref. < cT2)									
cT2	2.24	0.67–7.54	0.19	2.67	0.80–8.92	0.11	2.86	0.86–9.53	0.09
cT3	3.12	0.80–12.13	0.10	3.14	0.80–12.39	0.10	4.04	1.04–15.73	0.04
cT4	3.20	0.80–12.67	0.10	5.93	1.57–22.34	0.01	7.18	1.91–27.00	0.004
Radiologic suspected N+ prior Cx (Ref. N0)	2.14	0.93–4.92	0.07	1.71	0.76–3.82	0.19	1.39	0.63–3.07	0.41
High IL‐6 (Ref. low IL‐6 < 7 pg/mL)	4.14	2.10–8.14	<0.001	—	—	—	—	—	—
High GDF15 (Ref. low GDF15 < 1542 pg/mL)	—	—	—	2.23	1.04–4.80	0.04	—	—	—
Sarcopenia (Martin et al. Ref. no)	—	—	—	—	—	—	1.90	0.91–3.98	0.09

## 4 Discussion

To the best of our knowledge, this is the first study that elucidates the significant roles of IL‐6 and GDF‐15 in the context of sarcopenia among patients with BC undergoing RC. Our findings demonstrate that elevated preoperative serum levels of IL‐6 and GDF‐15 are closely associated with the presence of sarcopenia, adverse pathological features and diminished survival outcomes in patients undergoing RC for BC. These results underscore the potential of IL‐6 and GDF‐15 as prognostic biomarkers and therapeutic targets in this patient population.

The observed correlation between high IL‐6 levels and sarcopenia aligns with the existing literature. IL‐6 is a pro‐inflammatory cytokine implicated in muscle catabolism and cachexia, contributing to muscle wasting in patients with cancer [22, 23]. Sarcopenia alone was associated with worse tumour stages (*p* < 0.01; Figure S2 and Table S3). Existing studies in patients with BC and rectal cancer confirm this finding, but regardless of this connection, sarcopenia was shown to be an independent risk factor for survival in larger studies [2, 24]. In our study, sarcopenia was not quite identified as an independent predictor for OS and CSS; however, a statistical trend was shown (OS: HR 1.74, 95%CI 0.95–3.18, *p* = 0.07; CSS: HR 1.90, 95%CI 0.91–3.98, *p* = 0.09; Tables 4 and 5). Our limited patient count must be accounted for when interpreting these data. Elevated IL‐6 levels have been associated with increased muscle degradation and reduced muscle strength, thereby exacerbating sarcopenia [25]. In a meta‐analysis of 21 studies, Ding et al. found significantly elevated serum IL‐6 levels in adults with sarcopenia compared with those without sarcopenia (standard mean difference = 0.31; 95%CI 0.18–0.44) [26]. Similarly, we found higher serum IL‐6 values in patients with sarcopenia opposed to those without (*p* = 0.04; Figure 1). In multivariate analysis (Table 6), a trend was shown for high IL‐6 to be an independent predictor of sarcopenia (*p* = 0.08). Our study group has previously linked high IL‐6 expression to aggressive tumour behaviour and poor prognosis [8]. Other research suggested that IL‐6 may serve as a critical mediator connecting systemic inflammation, muscle wasting, and tumour progression [6, 27]. These findings can be confirmed by our present study showing the combined characteristics of high serum IL‐6 and sarcopenia opting for a significantly worse OS and CSS (log‐rank *p* < 0.01; Figure 2).

**TABLE 6:** Assessing biomarkers in the preoperative multivariate logistic regression model for sarcopenia including age, clinical tumour stage and radiologically suspected nodal stage prior to cystectomy.

Variable	Model 1 including IL‐6			Model 2 including GDF‐15		
	OR	(95%CI)	*p*	OR	(95%CI)	*p*
Age (years, continuous)	1.02	0.99–1.05	0.25	1.02	0.98–1.05	0.37
Clinical tumour stage prior to Cx (Ref. < cT2)						
cT2	1.56	0.69–3.50	0.29	1.50	0.66–3.38	0.33
cT3	1.17	0.39–3.49	0.78	1.06	0.35–3.23	0.91
cT4	1.51	0.38–6.00	0.56	1.74	0.45–6.69	0.42
Radiologic suspected N+ prior Cx (Ref. N0)	2.17	0.66–7.12	0.20	2.29	0.69–7.67	0.18
High IL‐6 (Ref. low IL‐6 < 7 pg/mL)	1.84	0.93–3.65	0.08	—	—	—
High GDF15 (Ref. low GDF15 < 1542 pg/mL)	—	—	—	1.99	1.00–3.96	0.05

The association between elevated GDF‐15 levels and sarcopenia observed in our study is supported by previous research [11, 28]. GDF‐15, a member of the transforming growth factor‐beta superfamily, is known to induce anorexia and weight loss, leading to muscle atrophy in patients with cancer [29]. Increased GDF‐15 expression has been correlated with poor clinical outcomes and reduced survival in various malignancies [30, 31]. In alignment with these findings, our study confirms high GDF‐15 levels in patients with worse OS and CSS in patients with BC (Figure 2C,D). We also found GDF‐15 to be an independent risk factor for OS and CSS in multivariate analysis (Tables 4 and 5). The combination of high GDF‐15 serum levels and the presence of sarcopenia was an independent risk factor for worse OS and CSS (Tables S5 and S6). Additionally, elevated serum levels of GDF‐15 are associated with unfavourable clinicopathologic tumour traits. For prostate cancer, several studies have shown an association between elevated serum GDF‐15 levels and bone metastasis [32, 33, 34]. Modi et al. found higher GDF‐15 levels in patients with breast cancer with larger tumour sizes (*p* = 0.018) and advanced tumour stages (*p* = 0.006) [35]. We also found high GDF‐15 levels to be associated with advanced tumour stages and positive surgical margins (Tables 1 and 3).

The clinical implications of our findings are multifaceted. Preoperative assessment of IL‐6 and GDF‐15 levels, alongside evaluation of sarcopenia, could enhance risk stratification and inform personalised treatment strategies. Interventions such as anti‐inflammatory therapies, nutritional support and tailored exercise programs may attenuate systemic inflammation and muscle wasting, potentially improving surgical outcomes and survival rates. Mouse models neutralising GDF‐15 or IL‐6 have confirmed the amelioration of muscle loss and therefore the potential for prolonged cancer survival [23, 28]. Specifically murine models in the context of cancer are also in focus of current research for both IL‐6 and GDF‐15 [36, 37, 38]. Examples of use in patients with cancer are still limited, and to this date, few studies exist that go beyond preclinical research. However, an example of successful treatment with Tocilizumab was given by Yu Dang et al. showing improved survival in patients with non‐small‐cell lung cancer [39]. Therefore, integrating IL‐6 and GDF‐15 inhibitors into therapeutic regimens warrants further investigation to disrupt the deleterious cycle of inflammation, muscle loss and tumour progression. Aiming to retrospectively identify patients who did not profit from surgery, and to explore whether the risk groups could prognosticate this, we identified 13 patients in our cohort who had tumour progression or metastasis within 12 months after surgery and died due to this cause. Of these 13 patients, 12 (92.3%) were in the high‐risk GDF‐15 group having both high GDF‐15 and sarcopenia. Given these findings in our retrospective analysis, almost all patients who died within 1 year due to cancer were in the high‐risk GDF‐15 group (high GDF‐15/sarcopenia). Nonetheless, we cannot conclude that patients in this risk group should not undergo surgery, as plenty of patients (73) were identified with CSS > 1 year, who also showed both traits (high GDF‐15/sarcopenia).

Our study is not free of limitations. The cohort size of 179 patients is limited and restricts the statistical analysis, especially regarding the extent of multivariate analyses. Also, our study is limited due to its single‐centre nature. A further limitation is the retrospective nature of the study, which limits consideration of confounding factors altering IL‐6 or GDF‐15 levels in serum. These may also include drug interactions or inflammatory diseases. The retrospective nature of the study also limits the information available on weight loss progression throughout cancer evolvement or already present cachexia at the time of primary resection. In the future, this should be taken into account for prospective studies. Additionally, certain findings are limited to univariate analysis. For instance, Figure 2 shows survival in the context of sarcopenia and levels of IL‐6 and GDF‐15 respectively. Secondary effects of sarcopenia, such as functional impairment, increased cardiovascular risk or malnutrition are not being considered in this analysis. Regarding statistical analysis, due to the exploratory nature of our study, no further adjustments were made to account for multiple comparisons; however, all results of univariate and multivariate analysis are presented in the supplementary information.

## 5 Conclusion

To the best of our knowledge, we presented the first study showing the prognostic significance of IL‐6 and GDF‐15 in relation to sarcopenia and adverse outcomes in patients undergoing RC for BC. High serum IL‐6 and GDF‐15 both were identified as independent risk factors for OS and CSS. In combination with sarcopenia, the risk for worse OS and CSS was even more prominent. Therefore, we were able to identify high‐risk patient groups prior to cystectomy by determining serum IL‐6, serum GDF‐15 and sarcopenia. Targeting these serum biomarkers presents a promising avenue for therapeutic intervention aimed at reducing sarcopenia and enhancing patient survival. Future research should focus on elucidating the precise molecular mechanisms underlying IL‐6 and GDF‐15‐mediated sarcopenia and evaluating the efficacy of targeted therapies in clinical trials.

## Author Contributions

Study concept and design: Engelmann, Mayr, Gužvić. Acquisition of data: Engelmann, Kasparbauer, Haas, Pickl, Rinderknecht. Analysis and interpretation of data: Engelmann, Mayr, Gužvić, Niessen, Siska, Del Giudice. Statistical analysis: Engelmann, Mayr, Rinderknecht. Drafting of the manuscript: Engelmann, Mayr. Critical revision of the manuscript for important intellectual content: All authors. Supervision: Burger, Gužvić, Mayr.

## Funding

The authors received no specific funding for this work.

## Ethics Statement

The study complies with the ethical standards described in the latest declaration of Helsinki; it was approved by the institutional ethics review board of the University of Regensburg (approval number: 08/108 and 24‐3636‐101).

## Conflicts of Interest

The authors declare no conflicts of interest.

## Supporting information


**Figure S1:** Scatter plots showing correlation between IL‐6 and GDF‐15. ‘A’ shows all data points and their correlation. ‘B’ shows only high IL‐6 and high GDF‐15 data points and their correlation. Red dotted lines indicate the cutoffs (IL‐6: 7 pg/mL, GDF‐15: 1541 pg/mL).
**Figure S2:** Bar graph showing number of patients (n) with different tumour stages grouped by the presence of sarcopenia.
**Table S1:** Showing all characteristics in different risk groups separated by sarcopenia and IL‐6 in serum.
**Table S2:** Showing all characteristics in different risk groups separated by sarcopenia and GDF‐15 in serum.
**Table S3:** Showing all characteristics in different risk groups separated by sarcopenia.
**Table S4:** Preoperative univariate cox regression analysis for overall survival (OS), cancer specific survival (CSS) and univariate logistic regression for Sarcopenia.
**Table S5:** Assessing IL‐6 and GDF‐15 risk groups in multivariate cox regression analysis for overall survival.
**Table S6:** Assessing IL‐6 and GDF‐15 risk groups in multivariate cox regression analysis for cancer‐specific survival.

## Data Availability

The datasets generated during and/or analysed during the current study are available from the corresponding author on reasonable request.
