# Supplementary material for: Interplay of IL‐6, GDF‐15 and Sarcopenia in Patients With Bladder Cancer Undergoing Radical Cystectomy and Its Implications on Survival
Source: J Cachexia Sarcopenia Muscle. 2025 Dec 11;16(6):e70146. doi: 10.1002/jcsm.70146 (PMC12698074; doi:10.1002/jcsm.70146)
Supplement: Supplementary file 1 — Figure S1: Scatter plots showing correlation between IL‐6 and GDF‐15. ‘A’ shows all data points and their correlation. ‘B’ shows only high IL‐6 and high GDF‐15 data points and their correlation. Red dotted lines indicate the cutoffs (IL‐6: 7 pg/mL, GDF‐15: 1541 pg/mL). Figure S2: Bar graph showing number of patients (n) with different tumour stages grouped by the presence of sarcopenia. Table S1: Showing all characteristics in different risk groups separated by sarcopenia and IL‐6 in serum. Table S2: Showing all characteristics in different risk groups separated by sarcopenia and GDF‐15 in serum. Table S3: Showing all characteristics in different risk groups separated by sarcopenia. Table S4: Preoperative univariate cox regression analysis for overall survival (OS), cancer specific survival (CSS) and univariate logistic regression for Sarcopenia. Table S5: Assessing IL‐6 and GDF‐15 risk groups in multivariate cox regression analysis for overall survival. Table S6: Assessing IL‐6 and GDF‐15 risk groups in multivariate cox regression analysis for cancer‐specific survival. [file JCSM-16-e70146-s001.docx]

# Supplementary Data

**Supplementary Figure 1.** Scatter plots showing correlation between IL-6 and GDF-15. ‘A’ showing all data points and their correlation. ‘B’ showing only high IL-6 and high GDF-15 data points and their correlation. Red dotted lines indicate the cut-offs (IL-6: 7 pg/ml, GDF-15: 1541 pg/ml)

**Supplementary Figure 2.** Bar graph showing number of patients (n) with different tumor stages grouped by the presence of sarcopenia.

**Supplementary Table 1.** Showing all characteristics in different risk groups separated by sarcopenia and IL-6 in serum.

| **Characteristic** | **No sarcopenia, low IL-6** | **Sarcopenia, low IL-6** | **No sarcopenia, High IL-6** | **Sarcopenia, High IL-6** | **p-value*** |
| --- | --- | --- | --- | --- | --- |
| Age (Median, IQR) | 68 (60-74.5) | 69.5 (62-76.8) | 69 (56-78.3) | 71 (62.8-81) |  |
| Gender |  |  |  |  | 0.53 |
| Male | 33 (80.5) | 47 (73.4) | 10 (62.6) | 41 (70.7) |  |
| Female | 8 (19.5) | 17 (26.6) | 6 (37.4) | 17 (29.3) |  |
| ASA-score |  |  |  |  | 0.15 |
| 1 | 1 (2.4) | 1 (1.6) | - | 1 (1.7) |  |
| 2 | 17 (41.5) | 30 (46.9) | 5 (31.3) | 18 (31) |  |
| 3 | 21 (51.2) | 33 (51.6) | 11 (68.8) | 32 (55.2) |  |
| 4 | 2 (4.9) | - | - | 7 (12.1) |  |
| Smoking |  |  |  |  | 0.64 |
| Yes | 23 (56.1) | 38 (59.4) | 8 (50) | 28 (48.3) |  |
| no | 18 (43.9) | 26 (40.6) | 8 (50) | 30 (51.7) |  |
| Neoadjuvant chemotherapy |  |  |  |  | 0.88 |
| Yes | 11 (26.8) | 18 (28.1) | 6 (37.5) | 17 (29.3) |  |
| No | 30 (73.2) | 46 (71.9) | 10 (62.5) | 41 (70.7) |  |
| Tumor stage at cystectomy |  |  |  |  | **<0.01** |
| pT0 | 11 (26.8) | 10 (15.6) | 2 (12.5) | 3 (5.2) |  |
| pTa, pT1, pTis | 8 (19.5) | 7 (10.9) | 3 (18.8) | 3 (5.2) |  |
| pT2 | 10 (24.4) | 19 (29.7) | 1 (6.3) | 3 (5.2) |  |
| pT3 | 10 (24.4) | 21 (32.8) | 8 (50) | 28 (48.3) |  |
| pT4 | 2 (4.9) | 7 (10.9) | 2 (12.5) | 21 (36.2) |  |
| Nodal stage |  |  |  |  | 0.12 |
| N0 | 34 (82.9) | 50 (78.1) | 13 (81.2) | 37 (63.8) |  |
| N+ | 7 (17.1) | 14 (21.9) | 3 (18.8) | 21 (36.2) |  |
| Surgical margins |  |  |  |  | **<0.01** |
| R0 | 37 (90.2) | 51 (79.7) | 11 (68.7) | 34 (58.6) |  |
| R+ | 4 (9.8) | 10 (15.6) | 5 (31.3) | 24 (41.4) |  |
| Rx | - | 3 (4.7) | - | - |  |
| Overall Death |  |  |  |  | **<0.01** |
| No | 33 (80.5) | 50 (78.1) | 10 (62.5) | 21 (36.2) |  |
| Yes | 8 (19.5) | 14 (21.9) | 6 (37.5) | 37 (63.8) |  |
| Cancer Specific Death |  |  |  |  | **<0.01** |
| No | 36 (87.8) | 55 (85.9) | 12 (75) | 30 (51.7) |  |
| Yes | 5 (12.2) | 9 (14.1) | 4 (25) | 28 (48.3) |  |

* Chi-squared statistic was used to determine differences between risk groups with respect to categorical variables

**Supplementary Table 2.** Showing all characteristics in different risk groups separated by sarcopenia and GDF-15 in serum.

| **Characteristic** | **No sarcopenia, low GDF-15** | **Sarcopenia, low GDF-15** | **No sarcopenia, High GDF-15** | **Sarcopenia, High GDF-15** | **p-value*** |
| --- | --- | --- | --- | --- | --- |
| Age (Median, IQR) | 66 (57-73) | 66 (59.5-75) | 70 (60.75-79.25) | 71 (65.5-79.5) |  |
| Gender |  |  |  |  | 0.95 |
| Male | 20 (74.1) | 26 (70.3) | 23 (76.7) | 62 (72.9) |  |
| Female | 7 (25.9) | 11 (29.7) | 7 (23.3) | 23 (27.1) |  |
| ASA-score |  |  |  |  | 0.06 |
| 1 | 1 (3.7) | 1 (2.7) | - | 1 (1.2) |  |
| 2 | 15 (55.6) | 20 (54.1) | 7 (23.3) | 28 (32.9) |  |
| 3 | 11 (40.7) | 16 (43.2) | 21 (70.0) | 49 (57.9) |  |
| 4 | - | - | 2 (6.7) | 7 (8.2) |  |
| Smoking |  |  |  |  | 0.13 |
| Yes | 14 (51.9) | 26 (70.3) | 17 (56.7) | 40 (47.1) |  |
| no | 13 (48.1) | 11 (29.7) | 13 (43.3) | 45 (52.9) |  |
| Neoadjuvant chemotherapy |  |  |  |  | 0.07 |
| Yes | 5 (18.5) | 6 (16.2) | 12 (40.0) | 29 (34.1) |  |
| No | 22 (81.5) | 31 (83.8) | 18 (60.0) | 56 (65.9) |  |
| Tumor stage at cystectomy |  |  |  |  | **<0.01** |
| pT0 | 10 (37.0) | 4 (10.8) | 3 (10.0) | 9 (10.6) |  |
| pTa, pT1, pTis | 3 (11.1) | 7 (18.9) | 8 (26.7) | 3 (3.5) |  |
| pT2 | 5 (18.5) | 12 (32.4) | 6 (20.0) | 10 (11.8) |  |
| pT3 | 7 (25.9) | 10 (27.0) | 11 (36.7) | 39 (45.9) |  |
| pT4 | 2 (7.4) | 4 (10.8) | 2 (6.7) | 24 (28.2) |  |
| Nodal stage |  |  |  |  | 0.09 |
| N0 | 24 (88.9) | 30 (81.1) | 23 (76.7) | 57 (67.1) |  |
| N+ | 3 (11.1) | 7 (18.9) | 7 (23.3) | 28 (32.9) |  |
| Surgical margins |  |  |  |  | **<0.01** |
| R0 | 23 (85.2) | 29 (78.4) | 25 (83.3) | 56 (65.9) |  |
| R+ | 4 (14.8) | 5 (13.5) | 5 (16.7) | 29 (34.1) |  |
| Rx | - | 3 (8.1) | - | - |  |
| Overall Death |  |  |  |  | **<0.01** |
| No | 22 (81.5) | 29 (78.4) | 21 (70.0) | 42 (49.4) |  |
| Yes | 5 (18.5) | 8 (21.6) | 9 (30.0) | 43 (50.6) |  |
| Cancer Specific Death |  |  |  |  | **0.02** |
| No | 24 (88.9) | 31 (83.8) | 24 (80.0) | 54 (63.5) |  |
| Yes | 3 (11.1) | 6 (16.2) | 6 (20.0) | 31 (36.5) |  |

* Chi-squared statistic was used to determine differences between risk groups with respect to categorical variables

**Supplementary Table 3.** Showing all characteristics in different risk groups separated by sarcopenia.

| **Characteristic** | **No sarcopenia** | **Sarcopenia** | **p-value*** |
| --- | --- | --- | --- |
| Age (Median, IQR) | 68 (60-75.5) | 70 (62-78) |  |
| Gender |  |  | 0.64 |
| Male | 43 (75.4) | 88 (72.1) |  |
| Female | 14 (24.6) | 34 (27.9) |  |
| ASA-score |  |  | 0.93 |
| 1 | 1 (1.8) | 2 (1.6) |  |
| 2 | 22 (38.6) | 48 (39.3) |  |
| 3 | 32 (56.1) | 65 (53.3) |  |
| 4 | 2 (3.5) | 7 (5.7) |  |
| Smoking |  |  | 0.97 |
| Yes | 31 (54.4) | 66 (54.1) |  |
| no | 26 (45.6) | 56 (45.9) |  |
| Neoadjuvant chemotherapy |  |  | 0.88 |
| Yes | 17 (29.8) | 35 (28.7) |  |
| No | 40 (70.2) | 87 (71.3) |  |
| Tumor stage at cystectomy |  |  | **<0.01** |
| pT0 | 13 (22.8) | 13 (10.7) |  |
| pTa, pT1, pTis | 11 (19.3) | 10 (8.2) |  |
| pT2 | 11 (19.3) | 22 (18.0) |  |
| pT3 | 18 (31.6) | 49 (40.2) |  |
| pT4 | 4 (7.0) | 28 (23.0) |  |
| Nodal stage |  |  | 0.11 |
| N0 | 47 (82.5) | 87 (71.3) |  |
| N+ | 10 (17.5) | 35 (28.7) |  |
| Surgical margins |  |  | 0.09 |
| R0 | 48 (84.2) | 85 (69.7) |  |
| R+ | 9 (15.8) | 34 (27.9) |  |
| Rx | - | 3 (2.5) |  |
| Overall Death |  |  | **0.03** |
| No | 43 (75.4) | 71 (58.2) |  |
| Yes | 14 (24.6) | 51 (41.8) |  |
| Cancer Specific Death |  |  | **0.04** |
| No | 48 (84.2) | 85 (69.7) |  |
| Yes | 9 (15.8) | 37 (30.3) |  |
| High IL-6 (cutoff 7pg/ml) |  |  | **0.02** |
| High | 16 (28.1) | 58 (47.5) |  |
| Low | 41 (71.9) | 64 (52.5) |  |
| High IL-6 (cutoff median 5.4pg/ml) |  |  | **0.04** |
| High | 22 (38.6) | 55 (45.1) |  |
| Low | 35 (61.4) | 67 (54.9) |  |

* Chi-squared statistic was used to determine differences between risk groups with respect to categorical variables

**Supplementary Table 4.** Preoperative univariate cox regression analysis for overall survival (OS), cancer specific survival (CSS) and univariate logistic regression for Sarcopenia

|  | **OS** | | | **CSS** | | | **Sarcopenia** | | |
| --- | --- | --- | --- | --- | --- | --- | --- | --- | --- |
| **Variable** | **HR** | **(95%CI)** | **p-Value** | **HR** | **(95%CI)** | **p-Value** | **OR** | **(95%CI)** | **p-Value** |
| Age (years, continuous) | 1.04 | 1.01-1.06 | **0.01** | 1.02 | 0.99-1.05 | 0.23 | 1.02 | 0.99-1.05 | 0.18 |
| Gender (Ref. Male) | 1.18 | 0.69-2.02 | 0.54 | 0.96 | 0.47-1.96 | 0.91 | 1.16 | 0.56-2.39 | 0.69 |
| Clinical Tumor Stage prior to Cx (Ref.<cT2) |  |  |  |  |  |  |  |  |  |
| cT2 | 1.72 | 0.76-3.99 | 0.19 | 3.05 | 0.92-10.1 | 0.07 | 1.68 | 0.76-3.67 | 0.20 |
| cT3 | 2.48 | 0.96-6.40 | 0.06 | 4.15 | 1.1-15.68 | **0.04** | 1.43 | 0.51-4.04 | 0.50 |
| cT4 | 5.49 | 2.21-13.64 | **<0.001** | 9.31 | 2.56-33.94 | **<0.001** | 2.68 | 0.74-9.70 | 0.13 |
| Radiologic suspected N+ prior Cx (Ref. N0) | 1.61 | 0.82-3.16 | 0.17 | 1.90 | 0.87-4.09 | 0.10 |  |  |  |
| ASA-score (Ref. 1) |  |  |  |  |  |  |  |  |  |
| 2 | 0.74 | 0.10-5.57 | 0.77 | 0.44 | 0.06-3.45 | 0.44 | 1.10 | 0.09-12.76 | 0.94 |
| 3 | 1.52 | 0.21-11.04 | 0.68 | 0.89 | 0.12-6.58 | 0.91 | 1.07 | 0.09-12.23 | 0.96 |
| 4 | 8.96 | 1.12-71.9 | **0.04** | 4.33 | 0.50-37.69 | 0.18 | 1.75 | 0.10-30.84 | 0.70 |
| Smoker (Ref. no) | 0.56 | 0.34-0.91 | **0.02** | 0.57 | 0.32-1.02 | 0.06 | 1.01 | 0.54-1.89 | 0.98 |
| Diabetes (Ref. no) | 1.42 | 0.81-2.50 | 0.23 | 1.34 | 0.68-2.64 | 0.40 | 0.53 | 0.25-1.12 | 0.10 |
| Neoadjuvant Chemotherapy | 0.59 | 0.33-1.07 | 0.08 | 0.76 | 0.39-1.46 | 0.41 | 1.01 | 0.50-2.03 | 0.99 |
| IL-6 (pg/ml, continuous) | 1.00 | 1.00-1.00 | **0.003** | 1.00 | 1.00-1.00 | 0.38 | 1.04 | 1.01-1.08 | **0.02** |
| High IL-6 (Ref. low IL-6 <7pg/ml) | 3.93 | 2.34-6.60 | **<0.001** | 4.60 | 2.44-8.65 | **<0.001** | 2.32 | 1.18-4.58 | **0.02** |
| GDF15 (pg/ml, continuous) | 1.00 | 1.00-1.00 | **<0.001** | 1.00 | 1.00-1.00 | **<0.001** | 1.00 | 1.00-1.00 | 0.08 |
| High GDF15 (Ref. low GDF15 <1542 pg/ml) | 2.64 | 1.43-4.85 | **0.002** | 2.67 | 1.29-5.55 | **0.008** | 2.07 | 1.08-3.95 | **0.03** |
| Sarcopenia (Martin et al. Ref. no) | 1.90 | 1.05-3.43 | **0.03** | 2.14 | 1.03-4.43 | **0.04** | - | - | **-** |

**Supplementary Table 5.** Assessing IL-6 and GDF-15 risk groups in multivariate cox regression analysis for overall survival.

|  | **Model 1 IL-6 Risk groups** | | | **Model 2 GDF-15 Risk groups** | | |
| --- | --- | --- | --- | --- | --- | --- |
| **Variable** | **HR** | **(95%CI)** | **p-Value** | **HR** | **(95%CI)** | **p-Value** |
| Age (years, continuous) | 1.02 | 0.10-1.05 | 0.08 | 1.02 | 0.99-1.05 | 0.14 |
| Clinical Tumor Stage prior to Cx (Ref.<cT2) |  |  |  |  |  |  |
| cT2 | 1..32 | 0.58-3.03 | 0.51 | 1.53 | 0.67-3.49 | 0.31 |
| cT3 | 2.00 | 0.75-5.32 | 0.16 | 1.92 | 0.71-5.19 | 0.20 |
| cT4 | 2.23 | 0.82-6.10 | 0.12 | 3.60 | 1.39-9.34 | **0.008** |
| Radiologic suspected N+ prior Cx (Ref. N0) | 1.66 | 0.79-3.45 | 0.18 | 1.46 | 0.72-2.99 | 0.30 |
| IL-6 Risk groups (Ref. no sarcopenia, low IL-6) |  |  |  |  |  |  |
| Sarcopenia, low IL-6 | 1.08 | 0.45-2.58 | 0.87 | - | - | - |
| No sarcopenia, high IL-6 | 2.34 | 0.78-6.99 | 0.13 | - | - | - |
| Sarcopenia, high IL-6 | 3.99 | 1.81-8.81 | **<0.001** | - | - | - |
| GDF-15 Risk groups (Ref. no sarcopenia, low GDF15) |  |  |  |  |  |  |
| Sarcopenia, low GDF-15 | - | - | - | 1.34 | 0.43-4.18 | 0.62 |
| No sarcopenia, high GDF-15 | - | - | - | 1.85 | 0.61-5.65 | 0.28 |
| Sarcopenia, high GDF-15 | - | - | - | 2.97 | 1.15-7.70 | **0.03** |

**Supplementary Table 6.** Assessing IL-6 and GDF-15 risk groups in multivariate cox regression analysis for cancer specific survival.

|  | **Model 1 IL-6 Risk groups** | | | **Model 2 GDF-15 Risk groups** | | |
| --- | --- | --- | --- | --- | --- | --- |
| **Variable** | **HR** | **(95%CI)** | **p-Value** | **HR** | **(95%CI)** | **p-Value** |
| Age (years, continuous) | 1.02 | 0.99-1.05 | 0.17 | 1.02 | 0.99-1.06 | 0.21 |
| Clinical Tumor Stage prior to Cx (Ref.<cT2) |  |  |  |  |  |  |
| cT2 | 2.22 | 0.66-7.46 | 0.20 | 2.63 | 0.79-8.78 | 0.12 |
| cT3 | 3.21 | 0.83-12.47 | 0.09 | 3.09 | 0.78-12.28 | 0.11 |
| cT4 | 3.44 | 0.86-13.79 | 0.08 | 5.92 | 1.55-22.58 | **0.01** |
| Radiologic suspected N+ prior Cx (Ref. N0) | 1.95 | 0.84-4.52 | 0.12 | 1.66 | 0.74-3.72 | 0.22 |
| IL-6 Risk groups (Ref. no sarcopenia, low IL-6) |  |  |  |  |  |  |
| Sarcopenia, low IL-6 | 1.10 | 0.37-3.31 | 0.87 | - | - | - |
| No sarcopenia, high IL-6 | 2.60 | 0.67-10.09 | 0.17 | - | - | - |
| Sarcopenia, high IL-6 | 4.78 | 1.79-12.77 | **0.002** | - | - | - |
| GDF-15 Risk groups (Ref. no sarcopenia, low GDF15) |  |  |  |  |  |  |
| Sarcopenia, low GDF-15 | - | - | - | 1.66 | 0.40-6.84 | 0.48 |
| No sarcopenia, high GDF-15 | - | - | - | 2.02 | 0.49-8.28 | 0.33 |
| Sarcopenia, high GDF-15 | - | - | - | 3.43 | 1.02-11.56 | **0.047** |
